# Mental health and substance use evolution in Swiss ED residents: a 6-month prospective longitudinal single-center study

**DOI:** 10.1007/s11739-025-04095-y

**Published:** 2025-09-02

**Authors:** Noémie Parejas, Nicolas Beysard, Michael Saraga, Pierre-Nicolas Carron

**Affiliations:** 1 Department of Emergency Medicine, Lausanne University Hospital (CHUV), Rue du Bugnon 46, 1011 Lausanne, Switzerland; 2 Faculty of Biology and Medicine, University of Lausanne, Rue du Bugnon 21, 1011 Lausanne, Switzerland; 3 Service of Liaison Psychiatry, Lausanne University Hospital (CHUV), Avenue de Beaumont 23, 1011 Lausanne, Switzerland

**Keywords:** Mental health, Emergency medicine, Working conditions, Psychological well-being, Burnout, Substance use

## Abstract

The challenging nature of emergency medicine places residents at risk of psychological strain and unhealthy consumption habits. Research on the impact of emergency department (ED) work on residents' mental health, substance use, and lifestyle is scarce. This exploratory mixed methods longitudinal study assessed ED residents at Lausanne University Hospital from 2020 to 2022. Residents completed surveys at the beginning and end of a 6-month ED rotation, including validated tools, such as the Maslach Burnout Inventory and the Hospital Anxiety and Depression Scale, alongside direct questions addressing substance use and lifestyle. Semi-structured interviews were then conducted to explore residents’ experiences and coping mechanisms. Primary outcomes included changes in burnout, anxiety, depression, and substance use. Secondary outcome was to gather residents' experiences in ED. Of 47 residents recruited, 31 completed follow-up surveys. Emotional exhaustion and depersonalization scores, subscales of the Maslach Burnout Inventory, increased slightly (*p* = 0.0259 and *p* = 0.0064), while personal accomplishment remained stable. Anxiety scores decreased (*p* = 0.0068), depression scores worsened (*p* = 0.0185), and sleep quality declined (*p* = 0.0022). Substance use and personal factors, such as religious beliefs and personal development activities, remained stable over the 6-month period. Interviews highlighted themes including patient flow pressure, irregular shifts, and departmental atmosphere. After 6 months in the ED, residents experienced minor changes in mental health with limited clinical significance. Protective factors like senior peer support may buffer against adverse effects. Future research should explore these dynamics in diverse EDs and over longer periods to better understand the impact on residents' well-being.

**Graphical Abstract:**

**Figure:**
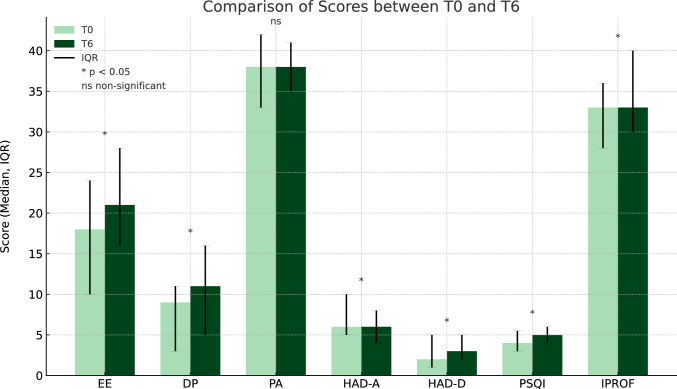

**Supplementary Information:**

The online version contains supplementary material available at 10.1007/s11739-025-04095-y.

## Introduction

For over 20 years, mental health disorders and at-risk consumption habits among physicians have emerged as a public health issue in Western countries. Recent studies reveal a high prevalence of psychological disorders among physicians due to workplace stress, including burnout, anxiety, depression, sleep disorders, post-traumatic stress disorder, and even suicide. These issues are also expressed through increased use of alcohol, medications (sleeping pills, painkillers), and drugs (marijuana, cocaine, heroin) [1–10]. Psychological distress among physicians is often underestimated because of its stigmatizing nature and their fear of career repercussions.

Emergency physicians are significantly affected by these issues, and psychological distress in emergency medicine is directly related to the specific work context of hospital emergency departments (EDs). EDs provide 24/7 care, which means more night, weekend, and holiday shifts than other departments [11, 12]. The workload in EDs is particular, with fluctuating patient volumes and varying case severity [13]. Emergency physicians must quickly identify and prioritize problems with limited time and resources, requiring high concentration, frequent decision making, and multitasking. Patients’ behavior toward healthcare professionals is sometimes exacerbated by substance abuse or long waiting times, which can lead to verbal or physical violence [14–16]. Emergency physicians also face increasing pressure for "zero error," reflected in the rise of complaints and legal actions against them [17, 18]. Finally, they are often seen—and see themselves—as highly resilient and robust, which fosters a culture in which admitting weakness is difficult. This point is even reflected in professional ethics guidelines, such as the American College of Emergency Physicians *Code of Ethics*, which highlights the importance of competence and resilience [19]. This high level of exposure is particularly challenging, as most of the ED residents are young and relatively inexperienced, potentially making them more vulnerable to the demanding nature of the ED environment.

The consequences of exposure to stress can then have repercussions on various levels, both individual and institutional. Psychological distress is known to reduce empathy and professional investment, leading to absenteeism, sick leave, and professional retraining, which ultimately decrease care quality and increase healthcare costs [20]. Many studies have examined the prevalence and causes of psychological disorders among emergency physicians, but most provide only a single snapshot in time, and few studies have explored the evolution of mental health in physicians working in the ED during a specific training period.

This study is exploratory in nature and aims to evaluate the evolution of general well-being, mental health (specifically burnout, anxiety, and depression), and substance consumption habits (alcohol, drugs, and medications) among emergency department (ED) residents after six months of exposure to this high-stress environment. The primary outcome measures will include the changes in burnout scores, anxiety, depression levels, and substance consumption habits over the study period. Secondary outcomes will focus on factors, such as coping strategies and the role of institutional support in mitigating psychological distress.

## Methods

This prospective, quantitative and qualitative, longitudinal single-center study included residents who had just started their residency in the ED of Lausanne University Hospital, Switzerland. The ED at Lausanne University Hospital is an academic tertiary hospital and level I trauma center, with approximately 50,000 admissions in 2021 [21]. The ED staff comprises 45 residents with varying levels of experience, who are under the constant supervision of chief residents or senior physicians. Each shift, whether daytime (8 a.m.–5:30 p.m.), evening (2 p.m.–10:30 p.m.), or night (10 p.m.–8 a.m.), is staffed by 7 residents. Residents work in blocks of 1 to 5 consecutive days, including weekends, which means that 3 weekends a month are affected as a whole or in part by shifts. According to Swiss labor law and European directives (EWTD), residents’ programmed working hours are limited to 50 h per week, including regulated overtime and mandatory rest periods. In our department, work schedules are planned in three-month cycles to ensure a balanced distribution of night shifts and weekends among all residents.

The sample size was determined based on convenience sampling, as residents newly hired in the ED during the study period were invited to participate.

Previously to this study, residents had access to two main institutional resources for psychological support: the liaison psychiatry service available to the ED team and the hospital’s Occupational Health Service.

### Participants

All newly hired residents who started in the ED from January 11, 2020 to January 5, 2022, were emailed a survey link upon arrival. Exclusion criteria included previous ED residency, professional activity rates of less than 80%, period of employment in the ED of less than 6 months or taking over a month off due to illness or accident.

### Study procedure

Residents who agreed to participate completed a first survey (*T*0) (Appendix 1) concerning their socio-demographic characteristics, lifestyle, substance use, and psychological well-being. The following standardized tests were used: Maslach Burnout Inventory (MBI) (measuring emotional exhaustion, depersonalization, personal accomplishment) [22], Hospital Anxiety and Depression Scale (HAD) (measuring anxiety and depression) [23], Pittsburgh Sleep Quality Index (PSQI) [24], and Professional Self-Identity Questionnaire for health and social care professions [25]. A summary of the tests used is available in Appendix 3.

After 6 months, participants were invited to complete an identical follow-up survey (*T*6). The *T*0 survey was to be completed within 2 weeks of starting their job in the ED and the *T*6 survey during the 2 weeks before finishing the 6-month period. Answers were anonymized, and the research team did not have access to participants’ identities. At the end of *T*6, participants were asked if they agreed to take part in the qualitative part of the study. If selected, their survey answers and their identity were shared with the examiner for semi-structured interviews.

Among those who agreed to participate, semi-structured interviews were proposed to participants with different evolution profiles. A senior psychiatrist with experience in qualitative research conducted the interviews within 12 months after *T*6, using an inductive approach without predefined hypotheses. The interview explored the participant’s experience of the ED shift, its impact on their professional and personal life (notably, relationships with close ones, personal and professional self-esteem, mood and anxiety, professional identity), and exploring in detail specific clinical situations identified as significant by the participant. Meetings were audio-recorded, supplemented with written notes, and lasted 45–60 min.

At the end of both *T*0 and *T*6, in case of need, participants could request a consultation with a psychiatrist independent from the research team to discuss any potential difficulties related to their work. Investigators did not know who made such requests, and the psychiatric consultation content was not disclosed. The independent psychiatrist did not have access to the survey answers or the research interviews. It is important to note that the psychiatrist conducting these interviews and the independent psychiatrist available for clinical support were two distinct persons with separated roles.

### Data analysis

Quantitative data were analyzed with Stata Statistical Software Release 14.0 (College Station, TX, USA: Stata Corporation; 2014). Statistical analyses were done using the Wilcoxon signed-rank test for continuous variables and the McNemar test for binary variables. To account for multiple comparisons among psychological outcome variables, p values were corrected using the Benjamini–Hochberg procedure (False Discovery Rate). The audio-recorded semi-structured interviews were analyzed using an inductive qualitative descriptive approach, with the aim of producing an overall synthesis of participants’ narratives, summarized under a limited number of themes. Concretely, the interviewer took notes after each interview, recording the most salient themes and stories. He then listened repeatedly to the recordings in order to confirm or amend his initial impressions and further refine his understanding of the participants’ experience of the ED shift. Finally, he summarized his findings in six synthetic themes presented in the results section.

This study was approved by our institutional ethical committee (CER-VD 2020-00561).

## Results

**Fig. 1:**
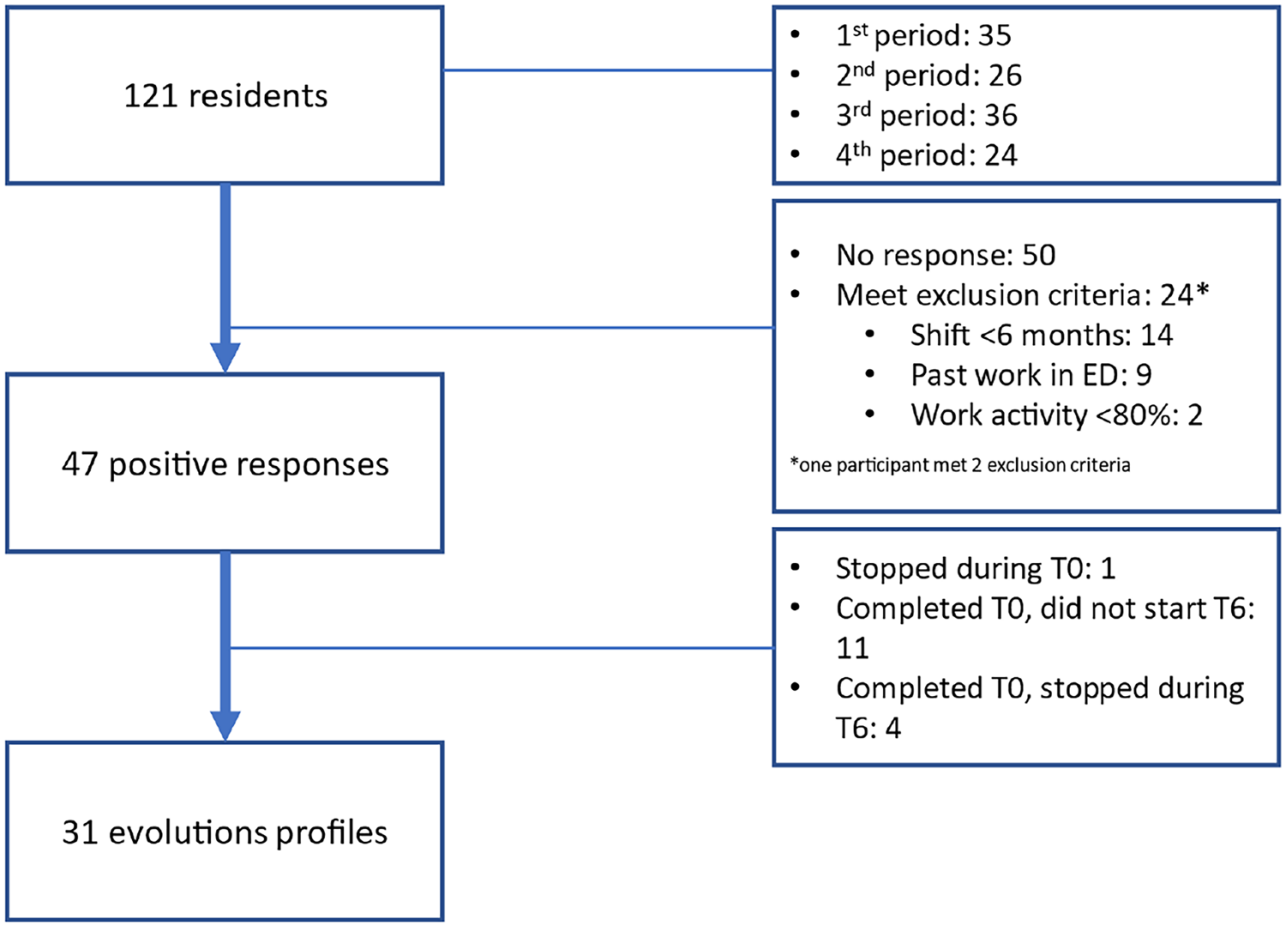
Flow chart. *T0*, baseline survey; *T6*, 6-month follow-up survey

**Fig. 2:**
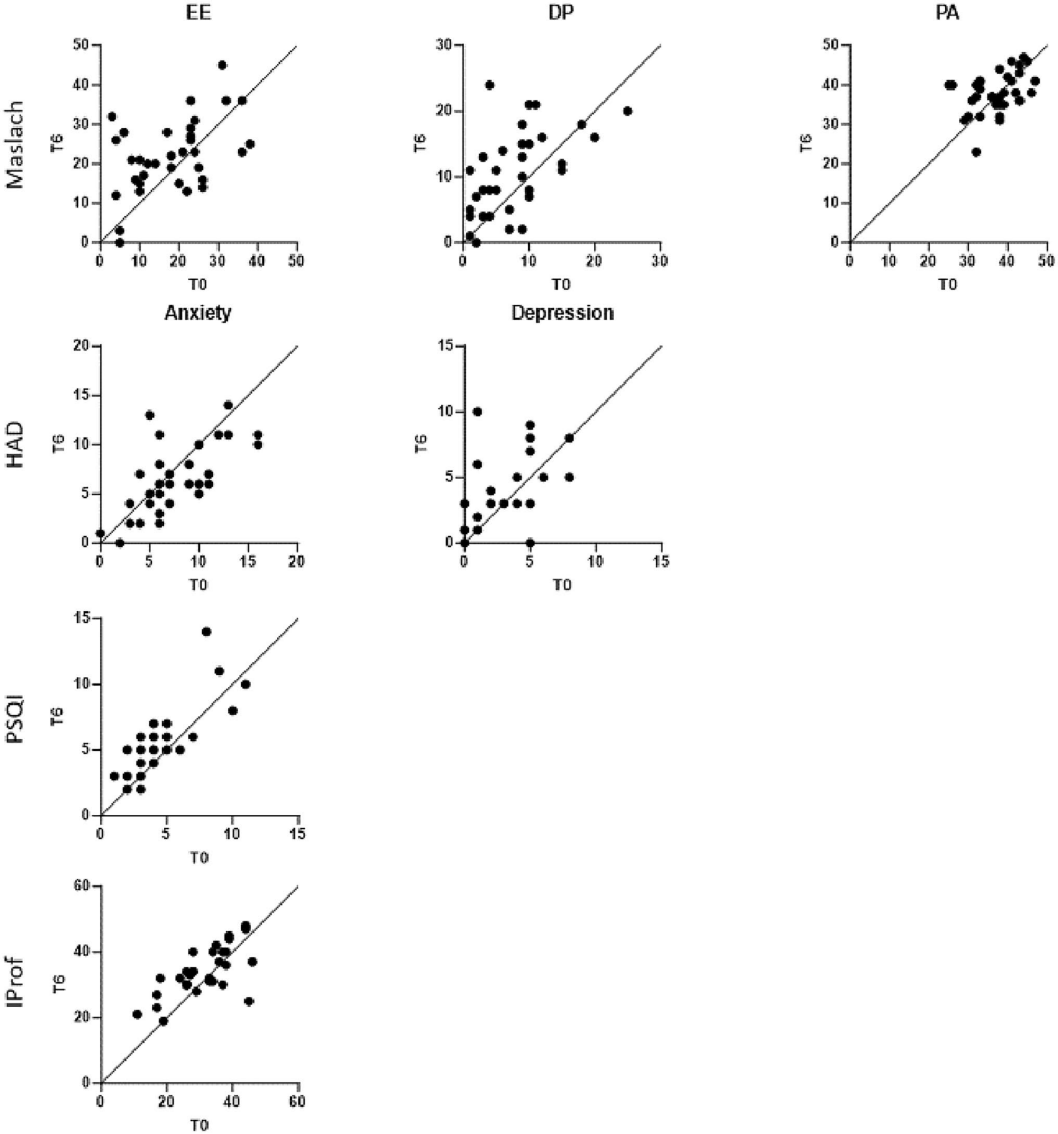
Evolution of quantitative analyses. *Maslach* Maslach Burnout Inventory score; *HAD* Hospital Anxiety and Depression Scale score; *PSQI* Pittsburgh Sleep Quality Index score; *IPROF* professional identity score; *T0* baseline survey; *T6* 6-month follow-up survey

From January 11, 2020 to January 5, 2022, 121 residents were invited to participate and 71 (58.7%) responded favorably. Among those who agreed to participate, 24 out of 71 (33.8%) met the exclusion criteria, leaving 47 out of 71 (66.2%) eligible for the *T*0 survey. 35 out of 71 (49.3%) also responded to the *T*6 survey. Four participants started but did not complete *T*6, resulting in 31 complete profiles. Flow-chart is available in Fig. 1. The partial answers of these 4 participants were, however, included in the statistical analyses. Answers to the survey given by all participants are summarized in Appendix 2. Comparisons of results between inclusion at T0 and T6 are presented in Table 1. The statistical evolution of the main scales tested is shown in Fig. 2. Participant’s demographic characteristics are available in Table 2. Six outcomes remained significant under FDR correction.

**Table 1:** Comparison of the various scores between T0 and T6

	*T*0	*T*6	*P*-value*	Cohen’s d**
EE, median (IQR) [0–54]^a^	18 (10–24)	21 (16–28)	**0.0259**	0.41
DP, median (IQR) [0–30]^a^	9 (3–11)	11 (5–16)	**0.0064**	0.50
PA, median (IQR) [0–48]^a^	38 (33–42)	38 (35–41)	0.5675	0.13
HAD-A, median (IQR) [0–21]^b^	6 (5–10)	6 (4–8)	**0.0068**	– 0.43
HAD-D, median (IQR) [0–21]^b^	2 (1–5)	3 (2–5)	**0.0185**	0.31
PSQI, median (IQR) [0–21]^c^	4 (3–5.5)	5 (4–6)	**0.0022**	0.61
IPROF, median (IQR) [9–54]^d^	33 (28–36)	33 (30–40)	**0.0046**	0.44
Alcohol, median (IQR) [units/week]^d^	3 (2–5)	2 (1–5)	0.0712	–
Tobacco users, number (%) [nbr]^d^	5 (16.1)	6 (19.3)	1.000	–
Tobacco consumption among users, median (IQR) [units/week]^d^	20 (20–35)	32.5 (30–35)	0.2188	–
Medication users, number (%) [nbr]^d^	6 (19.4)	8 (25.8)	0.6250	–
Drug users, number (%) [nbr]^d^	0 (0)	1 (3.1)	1.000	–
PDA, median (IQR) [hours/week]^d^	8 (5–14)	6 (4–10)	0.1353	–
Religion adherent, number (%) [nbr]^d^	3 (9.7)	4 (12.9)	1.000	–
PSY follow-up, number (%) [nbr]^d^	13 (41.9)	14 (45.2)	1.000	–

ED residents of Lausanne University hospital, 2020–2022

Partial answers of these 4 participants were included in the statistical analyses, leading to a different number of evolution profiles: a: *N* = 35; b: *N* = 33; c: *N* = 32; d: *N* = 31

*T0* baseline; *T6* 6-month follow-up; *ED* emergency department; [x–y] minimum and maximum possible scores for each scale;*IQR* interquartile range; *EE* emotional exhaustion; *DP* depersonalization; *PA* personal accomplishment; *HAD-A* Hospital Anxiety and Depression Scale_anxiety; *HAD-D* Hospital Anxiety and Depression Scale_depression; *PSQI* Pittsburgh Sleep Quality Index; *IPROF* professional identity score; *PDA* personal development activities; *nbr* number; *PSY* psychiatric

^*^P-values shown are uncorrected; all statistically significant results (*p* < .05) remained significant after Benjamini–Hochberg correction

^**^Cohen’s *d* was calculated only for continuous psychometric scores with paired data

Bold values indicate statistically significant differences ( p < 0.05).

**Table 2:** Characteristics of the study population (*n* = 35) consisting of ED residents who completed both surveys, Lausanne University Hospital, 2020–2022

Age in years, median (IQR)	28 (27–30)	
Male, *n* (%)	16 (45.7)	
Country of medical degree, *n* (%)		
Switzerland	23 (65.7)	
Italy	3 (8.6)	
Belgium	3 (8.6)	
Portugal	2 (5.7)	
France	1 (2.9)	
Spain	1 (2.9)	
Lebanon	1 (2.9)	
Morocco	1 (2.9)	
Previous experience in years, median (IQR)		
Total	2.5 (0.5–4)	
In Switzerland	2 (0–3)	
Previous experience in CHUV	0 (0–0.5)	
Work activity percentage, *n* (%)		
100%	30 (85.7)	
80%	5 (14.3)	
Planned specialty (-ies), *n*^**a**^		
Internal medicine^b^	20^b^	
Anesthesia	4	
Intensive care	1	
Cardiology	1	
Oncology	1	
General surgery	1	
Tropical medicine	1	
Nephrology	1	
Endocrinology	1	
Undefined	6	
Personal status, *n* (%)	T0	T6
In a relationship	19 (54.3)	17 (48.6)
Single	11 (31.4)	12 (34.3)
Married	5 (14.3)	6 (17.1)
Number of children, *n* (%)		
0	34 (97.1)	
2	1 (2.9)	

*IQR* interquartile range; *T0* baseline; *T6* 6-month follow-up

^a^Emergency medicine (ED) is not yet a specialty in Switzerland. It is additional training, which explains why it does not appear in the table. Most future emergency physicians pursue training in general internal medicine

^b^Among the residents considering a specialization in internal medicine, 6 were planning additional training in emergency medicine

Bold values indicate statistically significant differences ( p < 0.05).

Concerning the MBI subscale scores, emotional exhaustion (EE) increased (18, interquartile range [IQR] 10–24 vs. 21, IQR 16–28) after 6 months in the ED (*p* = 0.0259). High emotional exhaustion (≥ 30 points) was found in 5 out of 35 participants (14.3%) at *T*0 and 6 out of 35 (17.1%) at *T*6, the greatest individual increase being + 29 points. Depersonalization (DP) significantly increased (9, IQR 3–11 vs. 11, IQR 5–16) (*p* = 0.0064), with high depersonalization (≥ 12 points) in 8 out of 35 (22.8%) participants at *T*0 and 16 out of 35 (45.7%) at T6, the greatest individual increase being + 20 points. No significant difference in personal accomplishment was found between *T*0 and *T*6.

Regarding the HAD scale scores, the results showed that anxiety decreased after 6 months in the ED, despite identical median scores at *T*1 and *T*6 (6, IQR 5–10 vs. 6, IQR 4–8). Depression scores showed a slight but significant increase in median value (2, IQR 1–5 vs. 3, IQR 2–5) (*p* = 0.0185).

The PSQI scores indicated that sleep quality deteriorated over 6 months, with median scores increasing from 4 (IQR 3–5.5) to 5 (IQR 4–6) (*p* = 0.0022).

The Professional Self-Identity Questionnaire for health and social care professions showed a statistically significant increase in scores despite the same median score occurring at T0 and T6 (33, IQR 28–36 vs. 33, IQR 30–40) (*p* = 0.0046).

After 6 months, participants spent less time on personal development activities, averaging 8 (IQR 5–14) vs. 6 (IQR 4–10) hours per week. However, no significant changes were observed in alcohol, tobacco, medicine, or drug use, and the proportion of religious believers remained stable. 18 out of 31 participants (58%) showed minimal weight variation between T0 and T6 ( – 1.9 to + 1.9kg), the largest changes ranging from – 5kg (1 resident) to + 5kg (1 resident). At the first survey, 13 out of 31 residents (41.9%) had already consulted a mental health professional or were receiving follow-up care. By *T*6, this number had slightly increased to 14 out of 31 (45.2%).

Semi-structured interviews provided valuable insights into the unique aspects of emergency work and brought various subjects to light. A common stressor for participants was the pressure of patient flow. Although senior attending physicians oversee the management of overall flow, residents were highly concerned with waiting times and the number of patients in waiting rooms, and felt pressure to "move patients on." This pressure resulted in less time for each patient, inducing fear of medical errors and frustration from rushing their work. The lack of time sometimes led to a loss of empathy and reduced availability for therapeutic relationships. Overall, the pressure to manage flow contributed to a sense of practicing medicine that was misaligned with participants' values and beliefs.

The irregular working pattern had an impact on participants in various ways. Most felt out of sync with those around them, negatively affecting their social and family life. Some struggled with evening and night shifts, experiencing severe sleep issues (one participant had a car accident attributed to sleep deprivation). However, some participants appreciated the irregular rhythm and occasional weekdays off. Overall, irregular shifts were generally seen as negative, but there were notable exceptions.

Participants found emergency medicine's focus on diagnosing and treating acute problems satisfying. They appreciated the brief, punctual patient interactions, usually lasting a few hours. However, some were frustrated by the lack of involvement in patient follow-up during hospitalization. Overall, they valued the clinical aspects of emergency medicine, finding it more focused than was the case in other specialties.

Some participants shared deeply challenging emotional experiences in the ED, such as sudden unexpected deaths in young patients and breaking bad news in a rushed environment. These experiences did not cause major or lasting stress.

The ED ambiance was almost unanimously described as excellent and quite supportive. Participants appreciated the collaboration with the nursing team and with their peers, and they praised their supervisors as being highly competent, available, and very supportive.

## Discussion

To our knowledge, this is the first longitudinal study specifically examining the impact of 6 months of work in the ED on mental health and substance use among young emergency residents that was based on validated scores. Although similar longitudinal studies were conducted during the COVID-19 pandemic, they primarily focused on the mental health effects of the crisis rather than the specific challenges of working in the ED [26, 27]. This pandemic context may have further influenced outcomes, such as anxiety, sleep, and burnout, either amplifying or masking certain trends. This study was designed to evaluate the evolution of emergency residents in a usual context.

Our findings indicate statistically significant changes in several scores, including EE and DP. However, the associated effect sizes were consistently small to moderate (e.g., Cohen’s *d* = 0.41 for EE; Cohen’s *d* = 0.31 for HAD-D), suggesting limited clinical relevance. This interpretation is supported by participants’ nuanced reflections during the interviews, which revealed an overall positive perspective on their psychological state and an ability to contextualize stressors beyond what quantitative scores alone might convey. These clinical thresholds are consistent with the original validation studies and commonly used cut-offs in the literature [22–24]. However, as all outcomes were based on self-reported measures, social desirability bias cannot be ruled out, particularly for sensitive topics such as substance use or mental health symptoms.

The participants' initial emotional state and mental health were likely influenced by their previous work environments. At T0, the median EE score was 18 and DP was 9, indicating moderate burnout. Furthermore, 8 out of 35 participants (22.8%) presented HAD anxiety scores of ≥ 11, suggesting anxiety disorders, and 13 out of 32 (40.6%) had PSQI scores of ≥ 5, indicating significant sleep disturbances, even before being exposed to working in the ED. Although a direct comparison between our results and those in the literature is challenging due to the differences in populations and study designs, 2 meta-analyses provide relevant insights. The meta-analysis by Rotenstein et al. [3] focused on "medical students, resident physicians, and fellows," revealing a burnout prevalence ranging from 0 to 80.5%. Similarly, the review by Mata et al. [28] included only "resident physicians" and they estimated the depression prevalence in medical staff to be between 20.9 and 43.2%. However, these studies address a broader population of physicians and do not specifically focus on emergency physicians, making direct comparisons difficult.

Semi-structured interviews highlighted several key factors contributing to the stress experienced by residents, which may have influenced the observed changes in scores. Among these factors are the pressure from patient flow, which forces them to work quickly and increases the risk of making errors; irregular working hours that affect their personal lives; and exposure to emotionally intense situations.

The notable statistics that 13 out of 31 residents (41.9%) consulted a mental health professional in their lifetime raises important questions regarding mental health needs within this demographic. However, the study’s exploratory nature limited the ability to gather specific information on the reasons for these consultations, diagnoses, or follow-up frequency. Nonetheless, the 6 participants who sought help from an independent psychiatrist during the study highlighted the clear demand for mental health resources among residents.

Despite the challenges faced by the residents, several protective factors were identified during the interviews. They often emphasized the positive working environment and the adequate support they received from the emergency supervision team. According to the Copenhagen Psychosocial Questionnaire, an internationally recognized tool for assessing psychosocial risks in the workplace, support from supervisors and a favorable work atmosphere are crucial protective elements against the psychological toll of emergency work [29]. This suggests that the existing support framework may alleviate some of the stressors linked to working in the ED.

## Limitations

The small number of participants is the main limitation of this study. We aimed to recruit 60 participants over four 6-month periods, but recruitment varied by cohort: the presence of the main investigator during the introductory days of cohorts 2 and 3 likely boosted participation. The 6-month observation period reflects the average time residents spend in the ED, but longer follow-up might have revealed greater deterioration. In total, 47 participants responded at T0, with 35 remaining at T6, resulting in a dropout rate of about 25%. The reasons for this attrition are unclear but likely unrelated to the study. Although some loss to follow-up could introduce attrition bias, this is unlikely to have significantly influenced the results as the remaining participants showed consistent trends. However, it remains possible that the findings were slightly underestimated, especially if those lost to follow-up had different experiences. Finally, a post hoc power analysis indicated that 34 participants would be required to detect a moderate effect size (*d* = 0.5) with 80% power, suggesting that the final sample was slightly underpowered, particularly for detecting small effects, and that results should be interpreted with caution.

The quantitative part of the study was conducted from January 2020 to October 2022, overlapping entirely with the COVID-19 pandemic. Previous studies have shown that the pandemic can have a persistent and fluctuating impact on healthcare workers’ mental health [26, 27]. In our study, this context likely influenced outcomes, such as anxiety, sleep, and burnout. Without pre-pandemic baseline data, it is not possible to disentangle the specific impact of the pandemic, which limits the generalizability of our findings. This context should be considered as a potential confounding factor, and future research could compare pandemic and non-pandemic cohorts to better assess this impact.

Finally, a key limitation of this study is the sole reliance on self-reported measures. Social desirability bias is especially relevant among medical professionals and may have resulted in systematic underreporting of mental health symptoms or substance use [30]. Future research could benefit from including objective data, such as biometric indicators, absenteeism records, or peer assessments to complement self-reported outcomes. Moreover, the absence of a control group limits causal inference, as changes cannot be directly compared to residents in other settings.

## Conclusion

After six months of working in the ED, residents showed some statistically significant changes in their psychological scores. Emotional exhaustion increased by about 17% although most participants remained below the clinical burnout threshold. The proportion of residents reporting high depersonalization almost doubled from respectively 7 and 14 out to 31 (22.8% out to 46%), which may be of concern, while sleep quality also worsened and remained significant even after correction for multiple comparisons. However, the effect sizes for these changes were consistently small to moderate, and most scores stayed within ranges that do not indicate severe clinical deterioration. These results, together with participants’ nuanced perspectives shared during interviews, suggest that many residents are able to contextualize certain stressors and maintain an overall stable psychological state. Although this study focused on emergency medicine, similar stressors and irregular schedules are also found in other acute care specialties, which suggests that these findings may be relevant for residents in these fields as well.

From a practical standpoint, these findings underline the importance for ED leadership to maintain and reinforce protective measures, including accessible psychological support services and peer mentoring structures that appear to buffer stress, while also promoting awareness and use of existing institutional resources. Promoting good sleep hygiene could also help address emerging fatigue issues.

Future research should aim to validate these results using objective measures (e.g., actigraphy, work performance data), including relevant comparison groups, adopting multicenter designs to enhance generalizability, and extending follow-up beyond six months to assess the persistence and true clinical impact of these trends.

## Supplementary Information

Below is the link to the electronic supplementary material.

Supplementary file1 (DOCX 21 KB)

Supplementary file2 (DOCX 62 KB)

Supplementary file3 (DOCX 33 KB)

## Data Availability

The dataset supporting the conclusions of this article is available in Supplementary File 2.

## References

[1] Shanafelt TD, Boone S, Tan L, Dyrbye LN, Sotile W, Satele D et al (2012) Burnout and satisfaction with work-life balance among US physicians relative to the general US population. Arch Intern Med 172(18):1377–1385 22911330 10.1001/archinternmed.2012.3199

[2] Ma H, Huang SQ, We B, Zhong Y (2022) Compassion fatigue, burnout, compassion satisfaction and depression among emergency department physicians and nurses: a cross-sectional study. BMJ Open 12(4):e055941 35487521 10.1136/bmjopen-2021-055941 PMC9052053

[3] Rotenstein LS, Torre M, Ramos MA, Rosales RC, Guille C, Sen S et al (2018) Prevalence of burnout among physicians: a systematic review. JAMA 320(11):1131–1150 30326495 10.1001/jama.2018.12777 PMC6233645

[4] Kuhn G, Goldberg R, Compton S (2009) Tolerance for uncertainty, burnout, and satisfaction with the career of emergency medicine. Ann Emerg Med 54(1):106-113.e6 19201058 10.1016/j.annemergmed.2008.12.019

[5] KimoTakayesu J, Ramoska EA, Clark TR, Hansoti B, Dougherty J, Freeman W et al (2014) Factors associated with burnout during emergency medicine residency. Acad Emerg Med 21(9):1031–1035 25269584 10.1111/acem.12464

[6] Bragard I, Dupuis G, Fleet R (2015) Quality of work life, burnout, and stress in emergency department physicians: a qualitative review. Eur J Emerg Med 22(4):227–234 25093897 10.1097/MEJ.0000000000000194

[7] Estryn-Behar M, Doppia MA, Guetarni K, Fry C, Machet G, Pelloux P et al (2011) Emergency physicians accumulate more stress factors than other physicians–results from the French SESMAT study. Emerg Med J 28(5):397–410 21123828 10.1136/emj.2009.082594

[8] McBeth BD, Ankel FK, Ling LJ, Asplin BR, Mason EJ, Flottemesch TJ et al (2008) Substance use in emergency medicine training programs. Acad Emerg Med 15(1):45–53 18211313 10.1111/j.1553-2712.2007.00008.x

[9] Oreskovich MR, Shanafelt T, Dyrbye LN, Tan L, Sotile W, Satele D et al (2015) The prevalence of substance use disorders in American physicians. Am J Addict 24(1):30–38 25823633 10.1111/ajad.12173

[10] Hughes PH, Baldwin DC, Sheehan DV, Conard S, Storr CL (1992) Resident physician substance use, by specialty. Am J Psychiatry 149(10):1348–1354 1530071 10.1176/ajp.149.10.1348

[11] Papp KK, Stoller EP, Sage P, Aikens JE, Owens J, Avidan A et al (2004) The effects of sleep loss and fatigue on resident-physicians: a multi-institutional, mixed-method study. Acad Med 79(5):394–406 15107278 10.1097/00001888-200405000-00007

[12] Ferguson B, Shoff H, Shreffler J, McGowan J, Huecker M (2019) Does my emergency department doctor sleep? The trouble with recovery from night shift. J Emerg Med 57(2):162–167 31266687 10.1016/j.jemermed.2019.04.023

[13] Watson AG, McCoy JV, Mathew J, Gundersen DA, Eisenstein RM (2019) Impact of physician workload on burnout in the emergency department. Psychol Health Med 24(4):414–428 30372132 10.1080/13548506.2018.1539236

[14] de Boer J, Lok A, Van’tVerlaat E, Duivenvoorden HJ, Bakker AB, Smit BJ (2011) Work-related critical incidents in hospital-based health care providers and the risk of post-traumatic stress symptoms, anxiety, and depression: a meta-analysis. Soc Sci Med 73(2):316–326 21696873 10.1016/j.socscimed.2011.05.009 PMC7127421

[15] Schnapp BH, Slovis BH, Shah AD, Fant AL, Gisondi MA, Shah KH et al (2016) Workplace violence and harassment against emergency medicine residents. West J Emerg Med 17(5):567–573 27625721 10.5811/westjem.2016.6.30446 PMC5017841

[16] Howard L, Wibberley C, Crowe L, Body R (2018) How events in emergency medicine impact doctors’ psychological well-being. Emerg Med J 35(10):595–595 30131355 10.1136/emermed-2017-207218 PMC6173813

[17] Robertson JJ, Long B (2018) Suffering in silence: medical error and its impact on health care providers. J Emerg Med 54(4):402–409 29366616 10.1016/j.jemermed.2017.12.001

[18] Moberly T (2014) Rising complaints against doctors due to changed patient expectations, researchers say. BMJ 349:g4754 25099728 10.1136/bmj.g4754

[19] American College of Emergency Physicians. Code of Ethics for Emergency Physicians. Revised October 2023. Accessed 11 Aug 2024 https://www.acep.org/patient-care/policy-statements/code-of-ethics-for-emergency-physicians/

[20] Lu DW, Dresden S, McCloskey C, Branzetti J, Gisondi MA (2015) Impact of burnout on self-reported patient care among emergency physicians. West J Emerg Med 16(7):996–1001 26759643 10.5811/westjem.2015.9.27945 PMC4703144

[21] Les urgences, principale voie d’entrée au CHUV: rapport activité 2022. Accessed 14 Jan 2024. https://rapportsannuels.chuv.ch/activite/2022/1-3-les-urgences-principale-voie-dentree-au-chuv

[22] Maslach C, Jackson SE (1981) The measurement of experienced burnout. J Organ Behav 2(2):99–113

[23] Zigmond AS, Snaith RP (1983) The hospital anxiety and depression scale. Acta Psychiatr Scand 67(6):361–370 6880820 10.1111/j.1600-0447.1983.tb09716.x

[24] Buysse DJ, Reynolds CF, Monk TH, Berman SR, Kupfer DJ (1989) The Pittsburgh sleep quality index: a new instrument for psychiatric practice and research. Psychiatry Res 28(2):193–213 2748771 10.1016/0165-1781(89)90047-4

[25] Crossley J, Vivekananda-Schmidt P (2009) The development and evaluation of a professional self identity questionnaire to measure evolving professional self-identity in health and social care students. Med Teach 31(12):e603-607 19995162 10.3109/01421590903193547

[26] Hovland IS, Skogstad L, Stafseth S, Ekeberg Ø, Ræder J, Stafseth SK et al (2023) Prevalence of psychological distress in nurses, physicians and leaders working in intensive care units during the COVID-19 pandemic: a national one-year follow-up study. BMJ Open 13(12):e075190 38135308 10.1136/bmjopen-2023-075190 PMC10897841

[27] Müller MM, Baillès E, Blanch J, Torres X, Rousaud A, Cañizares S (2023) Burnout among hospital staff during the COVID-19 pandemic: longitudinal results from the international cope-corona survey study. J Psychosom Res 164:111102. 10.1016/j.jpsychores.2022.111102 36508846 10.1016/j.jpsychores.2022.111102 PMC9677553

[28] Mata DA, Ramos MA, Bansal N, Khan R, Guille C, Di Angelantonio E et al (2015) Prevalence of depression and depressive symptoms among resident physicians: a systematic review and meta-analysis. JAMA 314(22):2373–2383 26647259 10.1001/jama.2015.15845 PMC4866499

[29] Burr H, Berthelsen H, Moncada S, Nübling M, Dupret E, Demiral Y, International COPSOQ Network et al (2019) The third version of the Copenhagen psychosocial questionnaire. Saf Health Work 10(4):482–503. 10.1016/j.shaw.2019.10.002 31890332 10.1016/j.shaw.2019.10.002 PMC6933167

[30] Donaldson SI, Grant-Vallone EJ (2002) Understanding self-report bias in organizational behavior research. J Bus Psychol 17(2):245–262

