# Supplementary material for: Mental health and substance use evolution in Swiss ED residents: a 6-month prospective longitudinal single-center study
Source: Intern Emerg Med. 2025 Sep 2;21(5):1817–26. doi: 10.1007/s11739-025-04095-y (PMC13452789; doi:10.1007/s11739-025-04095-y)
Supplement: Supplementary file 1 [file 11739_2025_4095_MOESM1_ESM.docx]

# **Mental Health and Substance Use Evolution in Swiss ED Residents: A 6-Month Longitudinal Study**

# **Survey**

## **1. Demographic Data**

1.1. Age: (years)
1.2. Gender: (M/F/unspecified)
1.3. University of degree: (city and country)
1.4. Number of years of clinical experience: (n years)
1.5. Number of years of experience in Switzerland: (n years)
1.6. Number of years of experience at CHUV: (n years)
1.7. Current work activity rate: (%)
1.8. Planned specialty(ies): (answer/does not know)
1.9. Marital status: (married/divorced/widowed/in a relationship/single)
1.10. Children: (Yes/No)
🡪 1.10.1. If yes: number and age

## **2. Psychological well-being (standardized surveys)**

2.1. Maslach Burnout Inventory: The following items should be rated from 0 to 6 according to the following scale: 0 = never, 1 = a few times a year, 2 = once a month, 3 = a few times a month, 4 = once a week, 5 = a few times a week, 6 = daily

2.1.1. I feel emotionally drained by my work
2.1.2. I feel exhausted at the end of my workday
2.1.3. I feel tired when I wake up in the morning and have to face another day of work
2.1.4. I can easily understand what my patients are feeling
2.1.5. I feel that I treat some patients impersonally, as if they were objects
2.1.6. Working with people throughout the day requires a lot of effort from me
2.1.7. I handle my patients' problems very effectively
2.1.8. I feel like I'm breaking down because of my work
2.1.9. I feel that through my work, I have a positive influence on people
2.1.10. I have become more insensitive to people since I started this job
2.1.11. I fear that this job will make me emotionally hardened
2.1.12. I feel full of energy
2.1.13. I feel frustrated by my work
2.1.14. I feel like I work "too hard" in my job
2.1.15. I don't really care about what happens to some of my patients
2.1.16. Working in direct contact with people stresses me out too much
2.1.17. I can easily create a relaxed atmosphere with my patients
2.1.18. I feel refreshed when I have been close to my patients in my work
2.1.19. I have accomplished many worthwhile things in this job
2.1. 20. I feel burnt out
2.1.21. In my work, I deal with emotional problems very calmly
2.1.22. I feel that my patients hold me responsible for some of their problems

### 2.2. Hospital Anxiety and Depression Scale

2.2.1. I feel tense or nervous
0 = never, 1 = occasionally, 2 = often, 3 = most of the time
2.2.2. I take pleasure in the same things as before
0 = yes, as much as before, 1 = not as much, 2 = only a little, 3 = almost none
2.2.3. I have a feeling of fear as if something horrible is about to happen
0 = not at all, 1 = a little, but it doesn’t worry me, 2 = yes, but it’s not too bad, 3 = yes, very clearly
2.2.4. I laugh easily and see the bright side of things
0 = as much as in the past, 1 = not as much as before, 2 = really less than before, 3 = not at all
2.2.5. I worry
0 = very occasionally, 1 = occasionally, 2 = quite often, 3 = very often
2.2.6. I am in a good mood
0 = most of the time, 1 = quite often, 2 = rarely, 3 = never
2.2.7. I can sit quietly and do nothing and feel relaxed
0 = yes, no matter what, 1 = yes, generally, 2 = rarely, 3 = sometimes
2.2.8. I feel like I am functioning slowly
0 = never, 1 = sometimes, 2 = very often, 3 = almost always
2.2.9. I experience feelings of fear and have a knot in my stomach
0 = never, 1 = sometimes, 2 = quite often, 3 = very often
2.2.10. I am no longer interested in my appearance
0 = I am as interested as before, 1 = I may not pay as much attention, 2 = I don’t pay as much attention as I should, 3 = not at all
2.2.11 I can’t sit still
0 = not at all, 1 = not much, 2 = a little, 3 = yes, definitely
2.2.12. I look forward to doing certain things
0 = as much as before, 1 = a little less than before, 2 = much less than before, 3 = almost never
2.2.13. I experience sudden feelings of panic
0 = never, 1 = not very often, 2 = quite often, 3 = very often
2.2.14. I can enjoy a good book, radio program, or TV show
0 = often, 1 = sometimes, 2 = rarely, 3 = very rarely
2.3. Pittsburgh Sleep Quality Index: The following questions are about your sleep over the past month

2.3.1. What time do you go to bed? …
2.3.2. How many minutes does it take you to fall asleep? …
2.3.3. What time do you wake up? …
2.3.4. How many hours do you sleep on average? …
2.3.5. How many hours do you spend in bed on average?…

The following questions should be rated according to the following scale:
0 = not during the past month, 1 = less than once a week, 2 = once or twice a week, 3 = three times or more per week

2.3.6. During the past month, how often have you experienced sleep disturbances because you:
2.3.6.1. Couldn’t fall asleep after 30 minutes?
2.3.6.2. Woke up in the middle of the night or early in the morning?
2.3.6.3. Had to wake up to go to the bathroom?
2.3.6.4. Didn’t breathe comfortably?
2.3.6.5. Were coughing or snoring?
2.3.6.6. Felt too hot?
2.3.6.7. Felt too cold?
2.3.6.8. Had nightmares?
2.3.6.9. Had pain?
2.3.6.10. For the following reason (open field + frequency of the disturbance): …
2.3.7. During the past month, how often have you used medication, prescribed or not, to help you sleep? …
2.3.8. During the past month, how often have you had difficulty staying awake while driving, eating, or during social activities? …
2.3.9. During the past month, how much of a problem has it been for you to maintain enthusiasm for doing things? …
2.3.10. How would you rate your sleep over the past few months?
0 = very good, 1 = fairly good, 2 = fairly poor, 3 = very poor

2.4 Professional Identity: Regardless of your actual level of training, please evaluate how you currently feel about the following situations. The items should be rated from 1 to 6, with 1 corresponding to "I feel like a first-day resident" and 6 corresponding to "I feel like a newly qualified senior resident."

2.4.1. When I work with other health and social care professionals: 1 2 3 4 5 6
2.4.2. When I communicate with patients: 1 2 3 4 5 6
2.4.3. When I assess a patient: 1 2 3 4 5 6
2.4.4. When I am involved in the care of a patient with other healthcare professionals: 1 2 3 4 5 6
2.4.5. When I face ethical or moral questions: 1 2 3 4 5 6
2.4.6. When I consult a patient’s medical record: 1 2 3 4 5 6
2.4.7. When I face a patient-related emergency situation: 1 2 3 4 5 6
2.4.8. When I evaluate my practice to assess my learning needs: 1 2 3 4 5 6
2.4.9. When I teach others: 1 2 3 4 5 6

**3. Lifestyle and Consumption**

3.1. How many units of alcohol do you consume per week? (NOTE: one unit = 10g of alcohol, corresponding on average to 1 beer (3dl), 1 glass of wine (1dl), or 1 shot of hard liquor (0.4cl)): … units/week
3.2. How many cigarettes (or other tobacco products: cigars, cigarillos, e-cigarettes, hookah, etc.) do you smoke per week? …/week
3.3. Do you consume medications, with or without a prescription, for anxiolytic, relaxing, analgesic, recreational, or stimulating purposes? If yes, please specify:
- Substance(s): …
- Dosage: …
- Frequency of consumption: …/week
3.4. Do you use drugs? If yes, please specify:
- Type of substance: …
- Dosage: …
- Frequency of consumption: …/week
3.5. How many hours per week do you spend on activities that contribute to your personal well-being (e.g., sports, arts, hobbies, passions, family or social activities, etc.)? …hours/week
3.6. Do you feel connected to a spiritual or religious movement? Yes/No
3.7. Are you currently or have you ever been in psychological or psychiatric care? Yes/No
3.8. (second survey (T6) only) Since starting in the emergency department, have you noticed any fluctuation in your weight that is not attributable to a voluntary diet, a change in diet, illness, or pregnancy? Yes, …kg / No

**4. Conclusion**

4.1. First survey (T0) conclusion
Thank you for taking the time to respond! If, after completing this survey, you feel the need to discuss your professional situation with a senior psychiatrist independent from the research team, you may check the following box: …
The consultation is free. Your identity and your responses to the survey will not be disclosed to the research team.

4.2. Second survey (T6) conclusion
Thank you for taking the time to respond! If, after completing this survey, you feel the need to discuss your professional situation with a senior psychiatrist independent from the research team, you may check the following box: …
The consultation is free. Your identity and your answers to the surveys will not be disclosed to the research team.

The second part of the study will take the form of an individual interview. Please let us know if you would agree to be contacted for this stage. If you respond positively and your profile is selected, your identity, as well as the answers you provided in the survey, will be disclosed to the person conducting the interview (and only to that person): I accept / I refuse
