# Supplementary material for: Mental health and substance use evolution in Swiss ED residents: a 6-month prospective longitudinal single-center study
Source: Intern Emerg Med. 2025 Sep 2;21(5):1817–26. doi: 10.1007/s11739-025-04095-y (PMC13452789; doi:10.1007/s11739-025-04095-y)
Supplement: Supplementary file 2 [file 11739_2025_4095_MOESM2_ESM.docx]

|  | Participant 14 | | Participant 26 | | Participant 27 | | Participant 29 | | Participant 35 | | Participant 37 | | Participant 75 | |
| --- | --- | --- | --- | --- | --- | --- | --- | --- | --- | --- | --- | --- | --- | --- |
|  | T0 | T6 | T0 | T6 | T0 | T6 | T0 | T6 | T0 | T6 | T0 | T6 | T0 | T6 |
| **Maslach** |  |  |  |  |  |  |  |  |  |  |  |  |  |  |
| EE (0-54) | 16 | - | 9 | - | 16 | - | 10 | 21 | 4 | 26 | 21 | 23 | 18 | 22 |
| DP (0-30) | 11 | - | 12 | - | 4 | - | 5 | 8 | 3 | 13 | 1 | 11 | 10 | 7 |
| PA (0-48) | 37 | - | 39 | - | 43 | - | 43 | 36 | 46 | 38 | 40 | 42 | 32 | 40 |
| **HAD** |  |  |  |  |  |  |  |  |  |  |  |  |  |  |
| Anxiety (0-21) | 5 | - | 9 | - | 5 | - | 6 | 2 | 3 | 4 | 7 | 4 | 16 | 11 |
| Depression (0-21) | 1 | - | 0 | - | 4 | - | 0 | 0 | 1 | 6 | 2 | 3 | 5 | 7 |
| **PSQI (0-21)** | 2 | - | 9 | - | 1 | - | 2 | 2 | 4 | 4 | 6 | 5 | 4 | - |
| **Lifestyle** |  |  |  |  |  |  |  |  |  |  |  |  |  | - |
| Alcohol (U/week) | No | - | 5 | - | 1 | - | 5 | 3 | 3 | 0 | 1 | 0 | 0 | - |
| Tobacco (U/week) | 0 | - | 7 | - | 0 | - | 0 | 0 | 0 | 0 | 14 | 30 | 0 | - |
| Medication | No | - | No | - | No | - | No | No | No | No | Yes (fluoxetine) | No | Yes (lorazepam) | - |
| Drug | No | - | No | - | No | - | No | No | No | No | No | No | No | - |
| PDA (h/week) | 0 | - | 14 | - | 10 | - | 15 | 10 | 3 | 3 | 5 | 5 | 6 | - |
| Religion adherence | No | - | No | - | No | - | No | No | Yes | Yes | Yes | Yes | No | - |
| Psychiatric follow-up | Yes | - | No | - | No | - | Yes | Yes | Yes | Yes | No | Yes | Yes | - |
| Weight variation (kg) | X | - | X | - | X | - | X | 0 | X | 0 | X | +2 | X | - |
| **IPROF (9-54)** | 43 | - | 32 | - | 22 | - | 33 | 32 | 46 | 37 | 44 | 47 | 27 | - |
| **Need for psy support?** | - | - | - | - | - | - | - | - | - | - | - | - | - | - |
| **Semi-structured interviews** | X | - | X | - | X | - | X | OK | X | OK | X | OK | X | - |

EE: emotional exhaustion; DP: depersonalization; PA: personal accomplishment; HAD: Hospital Anxiety and Depression Scale; PSQI: Pittsburgh Sleep Quality Index; PDA: personal development activities; IPROF: professional identity score

|  | Participant 76 | | Participant 80 | | Participant 83 | | Participant 85 | | Participant 86 | | Participant 87 | | Participant 89 | |
| --- | --- | --- | --- | --- | --- | --- | --- | --- | --- | --- | --- | --- | --- | --- |
|  | T0 | T6 | T0 | T6 | T0 | T6 | T0 | T6 | T0 | T6 | T0 | T6 | T0 | T6 |
| **Maslach** |  |  |  |  |  |  |  |  |  |  |  |  |  |  |
| EE (0-54) | 24 | 23 | 24 | 31 | 4 | 12 | 20 | 15 | 23 | 26 | 23 | 36 | 14 | 20 |
| DP (0-30) | 10 | 21 | 5 | 11 | 6 | 14 | 9 | 15 | 1 | 5 | 11 | 21 | 10 | 8 |
| PA (0-48) | 41 | 41 | 33 | 39 | 44 | 47 | 37 | 35 | 33 | 32 | 38 | 31 | 30 | 32 |
| **HAD** |  |  |  |  |  |  |  |  |  |  |  |  |  |  |
| Anxiety (0-21) | 11 | 6 | 10 | 6 | 6 | 5 | 4 | 7 | 11 | 7 | 16 | 10 | 7 | 7 |
| Depression (0-21) | 6 | 5 | 0 | 3 | 2 | 4 | 3 | 3 | 8 | 8 | 1 | 2 | 1 | 1 |
| **PSQI (0-21)** | 6 | 5 | 3 | 6 | 2 | 5 | 3 | 2 | 8 | 14 | 4 | 6 | 4 | 5 |
| **Lifestyle** |  |  |  |  |  |  |  |  |  |  |  |  |  |  |
| Alcohol (U/week) | 3 | 10 | 3 | 2 | 3 | 2 | 3 | 2 | 0 | 0 | 2 | 3 | 1 | 0 |
| Tobacco (U/week) | 0 | 0 | 0 | 0 | 0 | 0 | 0 | 0 | 0 | 0 | 0 | 0 | 0 | 0 |
| Medication | No | No | No | No | No | Yes (Relaxane, Berocca) | No | No | Yes (tramadol) | Yes (Relaxane) | No | Yes (tizanidine) | No | No |
| Drug | No | No | No | No | No | No | No | No | No | No | No | No | No | No |
| PDA (h/week) | 5 | 5 | 10 | 6 | 3 | 2 | 8 | 10 | 7 | 6 | 15 | 15 | 3 | 5 |
| Religion adherence | No | No | No | No | No | No | No | No | Yes | Yes | No | No | No | No |
| Psychiatric follow-up | No | No | No | Yes | Yes | Yes | No | No | No | No | Yes | Yes | Yes | No |
| Weight variation (kg) | X | 0 | X | -2 | X | 0 | X | 0 | X | +2 | X | 0 | X | 0 |
| **IPROF (9-54)** | 35 | 42 | 18 | 32 | 33 | 31 | 39 | 45 | 26 | 30 | 26 | 34 | 39 | 44 |
| **Need for psy support?** | - | - | - | - | - | - | - | - | **X** | - | - | - | - | - |
| **Semi-structured interviews** | X | NO | X | OK | X | OK | X | NO | X | OK | X | OK | X | NO |

EE: emotional exhaustion; DP: depersonalization’ PA: personal accomplishment; HAD: Hospital Anxiety and Depression Scale; PSQI: Pittsburgh Sleep Quality Index; PDA: personal development activities; IPROF: professional identity score

|  | Participant 90 | | Participant 93 | | Participant 94 | | Participant 97 | | Participant 99 | | Participant 100 | | Participant 102 | |
| --- | --- | --- | --- | --- | --- | --- | --- | --- | --- | --- | --- | --- | --- | --- |
|  | T0 | T6 | T0 | T6 | T0 | T6 | T0 | T6 | T0 | T6 | T0 | T6 | T0 | T6 |
| **Maslach** |  |  |  |  |  |  |  |  |  |  |  |  |  |  |
| EE (0-54) | 17 | 28 | 3 | 32 | 18 | 19 | 32 | 36 | 20 | - | 38 | 25 | 23 | 27 |
| DP (0-30) | 12 | 16 | 1 | 4 | 9 | 18 | 15 | 11 | 15 | - | 20 | 16 | 7 | 5 |
| PA (0-48) | 26 | 40 | 47 | 41 | 32 | 37 | 38 | 32 | 37 | - | 36 | 37 | 39 | 38 |
| **HAD** |  |  |  |  |  |  |  |  |  |  |  |  |  |  |
| Anxiety (0-21) | 13 | 14 | 5 | 13 | 9 | 6 | 13 | 11 | 4 | - | 9 | 8 | 5 | - |
| Depression (0-21) | 5 | 9 | 1 | 2 | 4 | 3 | 4 | 5 | 2 | - | 8 | 5 | 2 | - |
| **PSQI (0-21)** | 11 | 10 | 2 | 3 | 5 | 5 | 5 | 7 | 7 | - | 10 | 8 | 5 | - |
| **Lifestyle** |  |  |  |  |  |  |  |  |  |  |  |  |  |  |
| Alcohol (U/week) | 1 | 0 | 0 | 0 | 7 | 8 | 10 | 2 | 0 | - | 4 | 2 | 1 | - |
| Tobacco (U/week) | 0 | 0 | 0 | 0 | 0 | 6 | 35 | 50 | 0 | - | 20 | 35 | 0 | - |
| Medication | Yes (melatonin) | Yes (melatonin) | No | No | No | No | Yes (dexamphetamine) | Yes (lisdexamfetamine) | Yes (melatonin) | - | No | No | No | - |
| Drug | No | No | No | No | No | Yes (cannabis) | No | No | No | - | No | No | No | - |
| PDA (h/week) | 10 | 1 | 25 | 3 | 10 | 10 | 25 | 30 | 3 | - | 18 | 10 | 0 | - |
| Religion adherence | No | No | No | Yes | No | No | No | No | Yes | - | No | No | Yes | - |
| Psychiatric follow-up | Yes | Yes | No | No | Yes | No | Yes | Yes | No | - | Yes | Yes | No | - |
| Weight variation (kg) | X | 0 | X | -2 | X | -4 | X | -5 | X | - | X | +5 | X | - |
| **IPROF (9-54)** | 34 | 40 | 34 | 31 | 17 | 27 | 29 | 28 | 38 | - | 37 | 30 | 42 | - |
| **Need for psy support?** | **X** | - | - | - | **X** | - | - | - | - | - | - | - | - | - |
| **Semi-structured interviews** | X | OK | X | NO | X | OK | X | NO | X | - | X | OK | X | - |

EE: emotional exhaustion; DP: depersonalization; PA: personal accomplishment; HAD: Hospital Anxiety and Depression Scale; PSQI: Pittsburgh Sleep Quality Index; PDA: personal development activities; IPROF: professional identity score

|  | Participant 104 | | Participant 105 | | Participant 107 | | Participant 108 | | Participant 109 | | Participant 111 | | Participant 115 | |
| --- | --- | --- | --- | --- | --- | --- | --- | --- | --- | --- | --- | --- | --- | --- |
|  | T0 | T6 | T0 | T6 | T0 | T6 | T0 | T6 | T0 | T6 | T0 | T6 | T0 | T6 |
| **Maslach** |  |  |  |  |  |  |  |  |  |  |  |  |  |  |
| EE (0-54) | 6 | - | 12 | 20 | 22 | 13 | 31 | 45 | 7 | - | 5 | 0 | 11 | 17 |
| DP (0-30) | 11 | - | 3 | 4 | 7 | 2 | 4 | 24 | 4 | - | 2 | 7 | 4 | 8 |
| PA (0-48) | 37 | - | 45 | 46 | 37 | 36 | 32 | 23 | 36 | - | 43 | 43 | 38 | 37 |
| **HAD** |  |  |  |  |  |  |  |  |  |  |  |  |  |  |
| Anxiety (0-21) | 6 | - | 6 | 2 | 12 | 11 | 6 | 8 | 4 | - | 3 | - | 4 | 2 |
| Depression (0-21) | 2 | - | 0 | 3 | 5 | 8 | 1 | 10 | 4 | - | 2 | - | 2 | 3 |
| **PSQI (0-21)** | 4 | - | 3 | 5 | 5 | 6 | 3 | 2 | 3 | - | 1 | - | 4 | 7 |
| **Lifestyle** |  |  |  |  |  |  |  |  |  |  |  |  |  |  |
| Alcohol (U/week) | 5 | - | 2 | 3 | 0 | 1 | 5 | 10 | 1 | - | 1 | - | 2 | 5 |
| Tobacco (U/week) | 0 | - | 0 | 0 | 0 | 0 | 50 | 35 | 0 | - | 0 | - | 0 | 0 |
| Medication | No | - | No | No | No | No | No | No | No | - | No | - | No | No |
| Drug | No | - | No | No | No | No | No | No | No | - | No | - | No | No |
| PDA (h/week) | 25 | - | 5 | 15 | 15 | 9 | 12 | 6 | 3 | - | 25 | - | 6 | 3 |
| Religion adherence | No | - | No | No | No | No | No | No | No | - | No | - | No | No |
| Psychiatric follow-up | No | - | No | No | Yes | Yes | No | Yes | No | - | No | - | No | No |
| Weight variation (kg) | X | - | X | 0 | X | +3 | X | 0 | X | - | X | - | X | +3 |
| **IPROF (9-54)** | 26 | - | 28 | 34 | 28 | 34 | 19 | 19 | 42 | - | 19 | - | 45 | 25 |
| **Need for psy support?** | - | - | - | - | - | - | - | - | - | - | - | - | - | - |
| **Semi-structured interviews** | X | - | X | OK | X | OK | X | OK | X | - | X | - | X | OK |

EE: emotional exhaustion; DP: depersonalization; PA: personal accomplishment; HAD: Hospital Anxiety and Depression Scale; PSQI: Pittsburgh Sleep Quality Index; PDA: personal development activities; IPROF: professional identity score

|  | Participant 116 | | Participant 118 | | Participant 120 | | Participant 123 | | Participant 124 | | Participant 127 | | Participant 129 | |
| --- | --- | --- | --- | --- | --- | --- | --- | --- | --- | --- | --- | --- | --- | --- |
|  | T0 | T6 | T0 | T6 | T0 | T6 | T0 | T6 | T0 | T6 | T0 | T6 | T0 | T6 |
| **Maslach** |  |  |  |  |  |  |  |  |  |  |  |  |  |  |
| EE (0-54) | 10 | 15 | 36 | 36 | 12 | 20 | 9 | 16 | 10 | - | 26 | 14 | 25 | 19 |
| DP (0-30) | 9 | 2 | 25 | 20 | 4 | 4 | 9 | 13 | 3 | - | 12 | 16 | 15 | 12 |
| PA (0-48) | 38 | 44 | 29 | 31 | 41 | 46 | 38 | 35 | 28 | - | 42 | 38 | 36 | 37 |
| **HAD** |  |  |  |  |  |  |  |  |  |  |  |  |  |  |
| Anxiety (0-21) | 6 | 6 | 10 | 10 | 3 | 2 | 6 | 3 | 5 | - | 7 | 6 | 5 | 5 |
| Depression (0-21) | 1 | 2 | 3 | 3 | 1 | 1 | 2 | 4 | 1 | - | 5 | 0 | 3 | 3 |
| **PSQI (0-21)** | 5 | 7 | 4 | 6 | 5 | 6 | 3 | 6 | 4 | - | 7 | 6 | 4 | 5 |
| **Lifestyle** |  |  |  |  |  |  |  |  |  |  |  |  |  |  |
| Alcohol (U/week) | 2 | 1 | 2 | 2 | 20 | 1 | 15 | 10 | 1 | - | 4 | 5 | 2 | 0 |
| Tobacco (U/week) | 20 | 30 | 0 | 0 | 0 | 0 | 0 | 0 | 0 | - | 0 | 0 | 0 | 0 |
| Medication | No | No | No | No | No | No | No | Yes (diphénhydramine) | No | - | Yes (lorazépam) | Yes (lorazépam) | No | No |
| Drug | No | No | No | No | No | No | No | No | No | - | No | No | No | No |
| PDA (h/week) | 10 | 10 | 15 | 4 | 4 | 5 | 7 | 6 | 4 | - | 5 | 6 | 2 | 3 |
| Religion adherence | No | No | No | No | No | No | No | No | Yes | - | No | No | No | No |
| Psychiatric follow-up | No | No | No | No | No | No | No | No | No | - | No | No | No | No |
| Weight variation (kg) | X | 0 | X | 0 | X | 0 | X | +2 | X | - | X | 0 | X | 0 |
| **IPROF (9-54)** | 28 | 40 | 27 | 33 | 37 | 40 | 36 | 37 | 25 | - | 38 | 36 | 26 | 30 |
| **Need for psy support?** | - | - | - | - | - | - | - | - | - | - | - | - | - | - |
| **Semi-structured interviews** | X | NO | X | NO | X | NO | X | OK | X | - | X | NO | X | OK |

EE: emotional exhaustion; DP: depersonalization; PA: personal accomplishment; HAD: Hospital Anxiety and Depression Scale; PSQI: Pittsburgh Sleep Quality Index; PDA: personal development activities; IPROF: professional identity score

|  | Participant 130 | | Participant 131 | | Participant 132 | | Participant 134 | | Participant 137 | | Participant 142 | | Participant 143 | |
| --- | --- | --- | --- | --- | --- | --- | --- | --- | --- | --- | --- | --- | --- | --- |
|  | T0 | T6 | T0 | T6 | T0 | T6 | T0 | T6 | T0 | T6 | T0 | T6 | T0 | T6 |
| **Maslach** |  |  |  |  |  |  |  |  |  |  |  |  |  |  |
| EE (0-54) | 6 | - | 6 | 28 | 23 | - | 5 | 3 | 26 | 16 | 8 | 21 | 24 | - |
| DP (0-30) | 9 | - | 2 | 0 | 16 | - | 1 | 1 | 3 | 8 | 12 | 16 | 4 | - |
| PA (0-48) | 37 | - | 43 | 36 | 31 | - | 43 | 45 | 25 | 40 | 39 | 35 | 20 | - |
| **HAD** |  |  |  |  |  |  |  |  |  |  |  |  |  |  |
| Anxiety (0-21) | 3 | - | 6 | 11 | 6 | - | 2 | 0 | 5 | 4 | 0 | 1 | 8 | - |
| Depression (0-21) | 0 | - | 2 | 3 | 3 | - | 0 | 0 | 0 | 1 | 0 | 1 | 3 | - |
| **PSQI (0-21)** | 3 | - | 6 | 5 | 4 | - | 1 | 3 | 2 | 5 | 1 | 3 | 6 | - |
| **Lifestyle** |  |  |  |  |  |  |  |  |  |  |  |  |  |  |
| Alcohol (U/week) | 3 | - | 8 | - | 3 | - | 1 | 1 | 2 | 1 | 7 | 7 | 30 | - |
| Tobacco (U/week) | 0 | - | 0 | - | 0 | - | 0 | 0 | 0 | 0 | 0 | 0 | 0 | - |
| Medication | No | - | No | - | No | - | No | No | No | No | No | No | No | - |
| Drug | No | - | No | - | No | - | No | No | No | No | No | No | No | - |
| PDA (h/week) | 5 | - | 10 | - | 5 | - | 10 | 15 | 5 | 6 | 10 | 6 | 2 | - |
| Religion adherence | No | - | Yes | - | No | - | No | No | No | No | No | No | No | - |
| Psychiatric follow-up | No | - | No | - | No | - | No | No | No | No | No | No | No | - |
| Weight variation (kg) | X | - | X | - | X | - | X | 0 | X | -2 | X | 0 | X | - |
| **IPROF (9-54)** | 36 | - | 38 | - | 20 | - | 44 | 48 | 38 | 40 | 11 | 21 | 33 | - |
| **Need for psy support?** | - | - | - | - | - | - | - | - | - | - | **X** | - | - | - |
| **Semi-structured interviews** | X | - | X | - | X | - | X | NO | X | OK | X | OK | X | - |

EE: emotional exhaustion; DP: depersonalization; PA: personal accomplishment; HAD: Hospital Anxiety and Depression Scale; PSQI: Pittsburgh Sleep Quality Index; PDA: personal development activities; IPROF: professional identity score

|  | Participant 149 | | Participant 150 | | Participant 152 | | Participant 155 | | Participant 157 | |
| --- | --- | --- | --- | --- | --- | --- | --- | --- | --- | --- |
|  | T0 | T6 | T0 | T6 | T0 | T6 | T0 | T6 | T0 | T6 |
| **Maslach** |  |  |  |  |  |  |  |  |  |  |
| EE (0-54) | 10 | - | 9 | - | 36 | 23 | 10 | 13 | 23 | 29 |
| DP (0-30) | 7 | - | 11 | - | 10 | 15 | 9 | 10 | 18 | 18 |
| PA (0-48) | 44 | - | 47 | - | 33 | 41 | 31 | 36 | 33 | 41 |
| **HAD** |  |  |  |  |  |  |  |  |  |  |
| Anxiety (0-21) | 4 | - | - | - | 11 | 6 | 5 | 4 | 10 | 5 |
| Depression (0-21) | 2 | - | - | - | 6 | 5 | 1 | 1 | 5 | 3 |
| **PSQI (0-21)** | 3 | - | - | - | 9 | 11 | 3 | 3 | 3 | 4 |
| **Lifestyle** |  |  |  |  |  |  |  |  |  |  |
| Alcohol (U/week) | 20 | - | - | - | 4 | 3 | 5 | 4 | 8 | 7 |
| Tobacco (U/week) | 60 | - | - | - | 0 | 0 | 0 | 0 | 0 | 0 |
| Medication | No | - | - | - | Yes (zolpidem) | Yes (zolpidem) | No | No | No | No |
| Drug | No | - | - | - | No | No | No | No | No | No |
| PDA (h/week) | 5 | - | - | - | 5 | 4 | 14 | 14 | 3 | 2 |
| Religion adherence | No | - | - | - | No | No | No | No | No | No |
| Psychiatric follow-up | No | - | - | - | Yes | Yes | Yes | Yes | Yes | Yes |
| Weight variation (kg) | X | - | X | - | X | -3 | X | +3 | X | 0 |
| **IPROF (9-54)** | 18 | - | - | - | 17 | 23 | 26 | 30 | 24 | 32 |
| **Need for psy support?** | - | - | - | - | - | - | **X** | - | **X** | - |
| **Semi-structured interviews** | X | - | X | - | X | No | X | OK | X | OK |

EE: emotional exhaustion; DP: depersonalization; PA: personal accomplishment; HAD: Hospital Anxiety and Depression Scale; PSQI: Pittsburgh Sleep Quality Index; PDA: personal development activities; IPROF: professional identity score
