# Supplementary material for: Mental health and substance use evolution in Swiss ED residents: a 6-month prospective longitudinal single-center study
Source: Intern Emerg Med. 2025 Sep 2;21(5):1817–26. doi: 10.1007/s11739-025-04095-y (PMC13452789; doi:10.1007/s11739-025-04095-y)
Supplement: Supplementary file 3 [file 11739_2025_4095_MOESM3_ESM.docx]

# Summary of Questionnaires and Interpretation Guidelines

The table below summarizes the validated surveys used in the study, the dimensions explored, the number of items, and provides interpretation guidelines to help understand score ranges where applicable.

| Survey | Acronym | Dimension Explored | Number of Items | Interpretation Guidelines |
| --- | --- | --- | --- | --- |
| Maslach Burnout Inventory | MBI | Burnout: Emotional Exhaustion (EE), Depersonalization (DP), Personal Accomplishment (PA) | 22 | EE: Low (<17), Moderate (17–26), High (≥27); DP: Low (<5), Moderate (5–9), High (≥10); PA: High (>39), Moderate (32–38), Low (<32) |
| Hospital Anxiety and Depression Scale | HAD | Anxiety (HAD-A), Depression (HAD-D) | 14 | Each subscale: 0–7 (normal), 8–10 (borderline), ≥11 (clinical case) |
| Pittsburgh Sleep Quality Index | PSQI | Sleep Quality | 19 | Global PSQI >5 indicates poor sleep quality |
| Professional Self Identity Questionnaire | IPROF | Professional Self-Identity | 9 | No established clinical threshold; higher scores indicate stronger professional identity |
